# Acquired Uterine Arteriovenous Fistulas After First-Trimester Pregnancy Loss: A Narrative Review with Case-Based Insights into Minimally Invasive Management

**DOI:** 10.3390/medicina62020422

**Published:** 2026-02-23

**Authors:** Răzvan-Grigoraș Căpitănescu, Elena-Iuliana-Anamaria Berbecaru, Anca-Maria Istrate-Ofițeru, Marian-Valentin Zorilă, Doru-Andrei Mitroi, Gabriela-Camelia Roșu, Dominic-Gabriel Iliescu, Roxana-Cristina Drăgușin, Laurențiu-Mihai Dîră, Maria-Cristina Comănescu, George-Lucian Zorilă

**Affiliations:** 1 Department of Obstetrics and Gynecology, Emergency County Hospital of Craiova, 200642 Craiova, Romania; razvan.capitanescu@umfcv.ro (R.-G.C.); mdoru95@yahoo.com (D.-A.M.); roxana.dragusin@umfcv.ro (R.-C.D.); lucian.zorila@umfcv.ro (G.-L.Z.); 2 Department of Obstetrics and Gynecology, University of Medicine and Pharmacy of Craiova, 200349 Craiova, Romania; laurentiu.dira@umfcv.ro; 3 Doctoral School, University of Medicine and Pharmacy of Craiova, 200349 Craiova, Romania; elena.berbecaru@umfcv.ro; 4 Department of Histology, Faculty of Medicine, University of Medicine and Pharmacy of Craiova, 200349 Craiova, Romania; camelia.rosu@umfcv.ro; 5 Research Centre for Microscopic Morphology and Immunology, University of Medicine and Pharmacy of Craiova, 200349 Craiova, Romania; 6 Department of Forensic Medicine, University of Medicine and Pharmacy of Craiova, 200349 Craiova, Romania; valentin.zorila@umfcv.ro; 7 Department of Anatomy, University of Medicine and Pharmacy of Craiova, 200349 Craiova, Romania; maria.comanescu@umfcv.ro

**Keywords:** uterine arteriovenous fistula, uterine arteriovenous malformation, post-abortion hemorrhage, color Doppler ultrasonography, uterine artery embolization, hysteroscopy, fertility-preserving treatment

## Abstract

*Background and Objectives*: Uterine arteriovenous fistulas (AVFs) and arteriovenous malformations (AVMs) are rare but potentially life-threatening vascular anomalies that most commonly develop after pregnancy-related uterine trauma, such as curettage or surgical evacuation. The widespread use of color Doppler ultrasonography has led to increased recognition of these lesions and a shift from hysterectomy toward fertility-preserving, minimally invasive management. This narrative review summarizes current evidence on acquired uterine AVF/AVM after early pregnancy loss, with particular emphasis on diagnostic challenges and contemporary therapeutic approaches, illustrated by representative clinical experience. *Materials and Methods*: A narrative review of the literature was conducted focusing on the pathophysiology, ultrasound and Doppler diagnostic criteria, interventional radiologic techniques, hysteroscopic management, and fertility outcomes in acquired uterine AVF/AVM. Illustrative clinical insights from anonymized post-abortion cases managed at our institution were incorporated solely to contextualize diagnostic and therapeutic considerations. *Results*: Color and spectral Doppler ultrasonography emerged as the diagnostic cornerstone, typically demonstrating serpiginous myometrial vessels with high-velocity, low-resistance turbulent flow, allowing for differentiation from retained products of conception. Uterine artery embolization showed high efficacy in achieving hemorrhage control, while hysteroscopic coagulation or resection represented an effective complementary or, in selected focal lesions, definitive treatment. Clinical experience highlighted that AVF-related vascularity may be evident early or may evolve over time, underscoring the importance of repeat Doppler evaluation in patients with persistent or recurrent bleeding. *Conclusions*: Acquired uterine AVF should be considered in women presenting with ongoing or recurrent uterine bleeding following aspiration abortion or curettage, even when initial Doppler findings are inconclusive. Individualized, minimally invasive strategies—often combining uterine artery embolization and hysteroscopic techniques—offer effective, uterus-preserving alternatives to hysterectomy.

## 1. Introduction

Uterine arteriovenous fistulas (AVFs) and arteriovenous malformations (AVMs) are uncommon but potentially life-threatening vascular anomalies of the uterus. They are characterized by abnormal, high-flow, low-resistance communications between uterine arteries and veins that bypass the capillary network and predispose affected women to severe uterine hemorrhage [1]. Although AVFs/AVMs may occur spontaneously, most cases encountered in clinical practice are acquired and develop following pregnancy-related events or iatrogenic uterine trauma, including dilation and curettage, cesarean delivery, myomectomy, and other intrauterine procedures [1,2,3]. In uterine vascular anomalies, the terms arteriovenous fistula (AVF) and arteriovenous malformation (AVM) are often used inconsistently in the clinical literature. In general, an AVF refers to a more focal direct communication between an artery and a vein, typically acquired and post-traumatic or iatrogenic in nature, whereas an AVM implies a more complex nidus-like vascular network with multiple feeding arteries and draining veins, often of congenital origin. In pregnancy-related acquired uterine lesions diagnosed primarily by ultrasound rather than angiographic mapping, this distinction may be blurred, and many reports use the term “AVM” as an umbrella designation. In this narrative review, we therefore use the combined term “acquired uterine AVF/AVM” to reflect this overlap, while specifying focal AVF-like lesions when hysteroscopic accessibility or localized shunting is suggested.

Historically regarded as exceptional entities, uterine AVFs/AVMs are being diagnosed with increasing frequency, largely due to the widespread use of transvaginal ultrasound combined with color and spectral Doppler imaging. These techniques allow for non-invasive visualization of abnormal myometrial vascularity and have led to greater recognition of acquired AVF/AVM and related conditions such as enhanced myometrial vascularity among women evaluated for abnormal uterine bleeding [1,3,4,5].

First-trimester aspiration abortion is a safe procedure, with major complication rates below 1% in large contemporary series [6,7]. Nevertheless, persistent or recurrent bleeding following abortion requires careful evaluation. The differential diagnosis includes retained products of conception, endometritis, gestational trophoblastic disease, and, less commonly, acquired uterine AVFs/AVMs [4,5,8,9]. Accurate diagnosis using color and spectral Doppler ultrasonography is essential, as repeat blind curettage in the presence of an unrecognized arteriovenous shunt may precipitate massive, potentially life-threatening hemorrhage [3,8].

Diagnosis relies primarily on transvaginal ultrasound with color and spectral Doppler, which typically demonstrates serpiginous anechoic channels within the myometrium and/or endometrium, associated with turbulent high-velocity flow and low-resistance indices [1,6]. Magnetic resonance imaging (MRI) and computed tomography angiography may provide complementary anatomic information, particularly in complex or extensive lesions [4]. Over the past two decades, management strategies have evolved from routine hysterectomy toward fertility-preserving, minimally invasive approaches, including uterine artery embolization (UAE), conservative surgical techniques, and, more recently, hysteroscopic resection or coagulation of the malformation [5,10,11,12,13,14,15,16].

The aim of this narrative review is to synthesize current evidence on acquired uterine AVFs/AVMs, with particular emphasis on post-pregnancy lesions, diagnostic challenges, and contemporary fertility-preserving management strategies. Clinical insights derived from post-abortion cases managed with minimally invasive techniques, including UAE and hysteroscopy, are integrated to contextualize key diagnostic and therapeutic considerations.

To visually summarize the diagnostic pathway, key imaging features, and contemporary fertility-preserving management strategies discussed in this review, a graphical abstract is provided. This schematic highlights the central role of transvaginal ultrasound with color Doppler in identifying high-flow uterine arteriovenous shunts after first-trimester pregnancy loss, as well as the stepwise use of minimally invasive treatments—particularly uterine artery embolization and hysteroscopic intervention—in appropriately selected patients. The graphical abstract also emphasizes practical clinical recommendations aimed at avoiding misdiagnosis and preventing potentially life-threatening hemorrhage.

Compared with previously published reviews, the present narrative review provides a focused analysis of acquired uterine arteriovenous fistulas and malformations specifically in the context of first-trimester pregnancy loss and post-abortion bleeding. In addition to summarizing established diagnostic and therapeutic concepts, this review highlights several underemphasized aspects: (i) diagnostic pitfalls in the early post-abortion setting, including the temporal evolution of Doppler findings and the clinical relevance of repeat ultrasound evaluation; (ii) a pragmatic, minimally invasive management framework integrating uterine artery embolization and hysteroscopy while explicitly acknowledging the level and limitations of available evidence; and (iii) illustrative case-based imaging correlations that contextualize cavity-adjacent lesions potentially amenable to hysteroscopic management. By integrating current literature with carefully delineated clinical insights, this review aims to complement prior publications and provide practical, context-specific guidance without proposing practice-changing recommendations.

### Materials and Methods

This manuscript was designed as a non-systematic narrative review focusing on acquired uterine arteriovenous fistulas and malformations following early pregnancy loss. A literature search was conducted in PubMed/MEDLINE and cross-checked using the “related articles” function and citation tracking. The primary search term used was “uterine arteriovenous malformation”. The search covered the period from January 2000 to February 2026 and was last updated on 6 February 2026.

To ensure comprehensive coverage of diagnostic and therapeutic aspects, additional searches were performed using combinations of related keywords, including “uterine arteriovenous fistula”, “enhanced myometrial vascularity”, “post-abortion hemorrhage”, “retained products of conception”, “color Doppler”, “transvaginal ultrasound”, “uterine artery embolization”, and “hysteroscopy”. The primary PubMed search yielded 546 records.

Eligibility criteria included English-language publications reporting acquired uterine arteriovenous fistulas or malformations, or closely related high-flow uterine vascular anomalies, in the context of pregnancy-related uterine trauma or pregnancy loss, with relevant diagnostic, imaging, management, or outcome data. We excluded congenital pelvic arteriovenous malformations not centered in the uterus, vascular tumors, and reports unrelated to pregnancy or uterine instrumentation.

Titles and abstracts were screened for relevance, and full-text articles were reviewed when necessary. Reference lists of included articles and key review publications were manually screened to identify additional relevant studies. After screening and assessment of relevance, approximately 30 articles were included in the narrative synthesis.

Given the rarity of the condition and the predominance of case reports and small case series in the available literature, no formal systematic review methodology or quantitative quality assessment was performed. Instead, findings were synthesized narratively, and clinical considerations were interpreted in light of the level and limitations of the available evidence.

In addition to the literature synthesis, anonymized clinical images and qualitative observations from post-abortion cases managed at our institution were included solely for illustrative purposes, to contextualize diagnostic and therapeutic considerations. These institutional cases were not analyzed as a case series, and no statistical comparisons were performed. Written informed consent for anonymous participation and for the scientific use of clinical and imaging data was obtained from all participants. This study was approved by the Ethics Committee of the Craiova County Emergency Clinical Hospital (Approval No. 50162, 21 October 2025).

## 2. Acquired Uterine Arteriovenous Fistulas and Malformations: Current Evidence

### 2.1. Pathophysiology and Classification

Uterine arteriovenous fistulas and malformations are traditionally classified as congenital or acquired [2]. Congenital AVMs arise from aberrant embryologic development of the primitive vascular plexus and are typically characterized by complex vascular networks consisting of multiple feeding arteries and dilated draining veins. These lesions may be extensive and can involve not only the uterus but also adjacent pelvic vascular structures [2].

In contrast, acquired uterine AVFs/AVMs are encountered more frequently in clinical practice and usually develop following pregnancy-related events or iatrogenic uterine trauma, including dilation and curettage, cesarean delivery, and other intrauterine surgical procedures [1,2,3,9].

The pathogenesis of acquired lesions is thought to involve abnormal vascular healing and remodeling in areas previously affected by trophoblastic invasion or surgical injury. Disruption of the normal arteriolar–capillary–venular architecture may result in persistent direct arteriovenous communications, giving rise to fragile, high-flow vascular channels that fail to regress after pregnancy termination or delivery [1,3,4,9]. In some cases, retained products of conception or persistent trophoblastic tissue may further stimulate neovascularization and impair normal postpartum involution, leading to enhanced myometrial vascularity or the development of a true arteriovenous malformation [1,3,9].

### 2.2. Epidemiology and Risk Factors

Most patients reported with acquired uterine AVF/AVM are of reproductive age and have a recent history of pregnancy, miscarriage, induced abortion, or uterine surgery [1,3,4,9]. Systematic reviews indicate that antecedent uterine trauma is present in the majority of cases, with diagnostic or therapeutic curettage identified as one of the most frequent precipitating factors [3,9].

Pregnancy-related AVFs/AVMs have been described following spontaneous or induced abortion, cesarean section, manual removal of the placenta, and uterine artery embolization performed for postpartum hemorrhage [3,8,9]. Despite these recognized associations, the true incidence of acquired uterine AVFs/AVMs remains unknown, as a substantial proportion of cases are likely asymptomatic or underdiagnosed.

**Figure 1:**
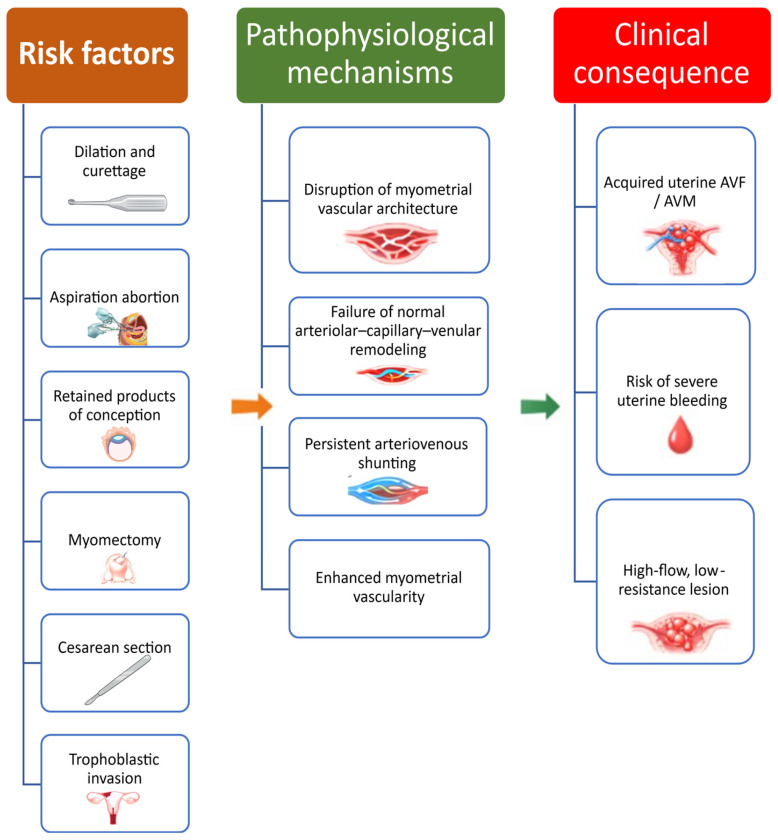
Schematic representation of risk factors, pathophysiological mechanisms, and clinical consequences of acquired uterine arteriovenous fistulas and malformations. Pregnancy-related uterine trauma and trophoblastic invasion may disrupt normal myometrial vascular remodeling, leading to persistent arteriovenous shunting, enhanced myometrial vascularity, and the development of high-flow uterine vascular lesions associated with severe uterine bleeding. This figure summarizes concepts discussed in Section 2.1 and Section 2.2. Created with BioRender. Istrate-Ofiteru, A. (2026) https://BioRender.com/g7rp8jz. (accessed on 10 February 2026).

Nevertheless, increasing clinical awareness and routine use of transvaginal color Doppler ultrasound have contributed to more frequent recognition of these lesions, particularly among women evaluated for abnormal uterine bleeding in the postpartum and post-abortion periods [1,3,4,9]. The main risk factors and pathophysiological mechanisms are summarized schematically in Figure 1.

### 2.3. Clinical Presentation

Abnormal uterine bleeding represents the predominant clinical manifestation of acquired uterine AVF/AVM. Clinical presentations range from prolonged spotting and intermittent heavy bleeding to sudden, massive hemorrhage with hemodynamic instability [1,2,3,4,9]. Consequently, anemia is frequently observed in patients with chronic or recurrent bleeding episodes.

The timing of presentation is variable. Some patients develop acute severe bleeding shortly after the inciting event, whereas others present weeks or months later [3,9]. Pelvic pain is inconsistent and may be absent. Given that symptoms overlap with more prevalent conditions—such as retained products of conception, subinvolution of the placental site, endometritis, or gestational trophoblastic disease—a high index of suspicion is warranted in women with persistent or recurrent bleeding following curettage, cesarean delivery, or aspiration abortion [1,3,9]. In some cases, uterine AVF/AVM is detected incidentally during imaging performed for infertility evaluation or other gynecologic indications [2,9].

### 2.4. Imaging Diagnosis

**Figure 2:**
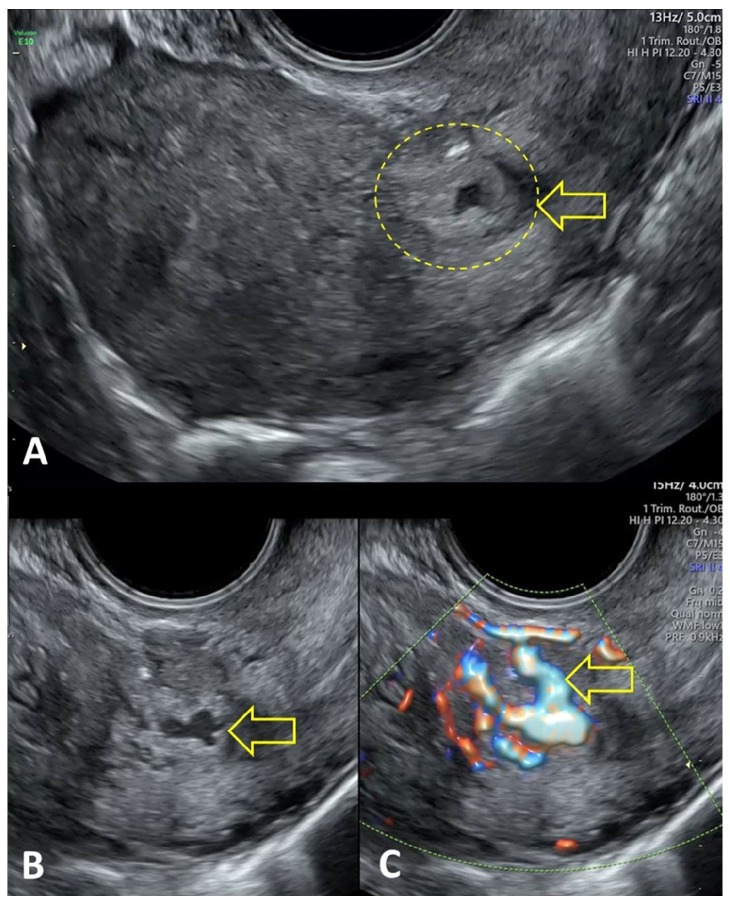
Transvaginal ultrasound and color Doppler findings characteristic of acquired uterine arteriovenous fistula/malformation (AVF/AVM). (**A**,**B**) Gray-scale transvaginal ultrasound showing a heterogeneous myometrium with clusters of serpiginous anechoic channels adjacent to a previous cesarean-section scar (indicated by a yellow arrow). (**C**) Color Doppler demonstrating intense aliasing and a mosaic pattern of turbulent, high-velocity, low-resistance flow consistent with arteriovenous shunting (indicated by a yellow arrow). AVF, arteriovenous fistula; AVM, arteriovenous malformation.

Transvaginal ultrasound is the first-line imaging modality for suspected uterine AVF/AVM. Although grayscale findings may be nonspecific, color and spectral Doppler ultrasound are essential for diagnosis. Characteristic Doppler findings include a tangle of vessels exhibiting a mosaic (“aliasing”) color pattern with multidirectional turbulent flow, as well as spectral waveforms demonstrating high peak systolic velocities and low-resistance indices consistent with arteriovenous shunting (Figure 2) [1,4]. When interpreted in the appropriate clinical context, these Doppler criteria strongly support the diagnosis of uterine AVF/AVM and aid in differentiation from other causes of abnormal uterine bleeding [1,3,4].

**Figure 3:**
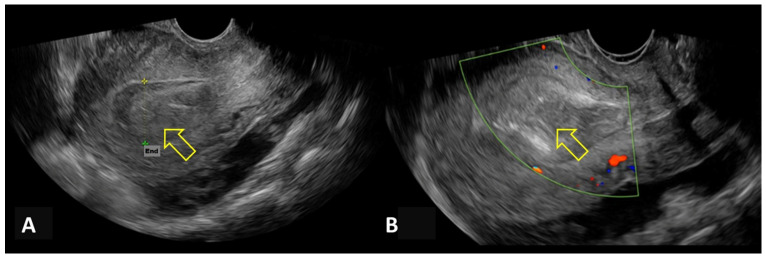
Initial transvaginal ultrasound examination in a patient with post-abortion bleeding. (**A**) Gray-scale ultrasound showing mixed hypo- and hyperechogenic material within the endometrial cavity, compatible with clot or necrotic tissue (indicated by a yellow arrow); End: endometrial thickness. (**B**) Absence of abnormal Doppler flow on initial evaluation (indicated by a yellow arrow).

**Figure 4:**
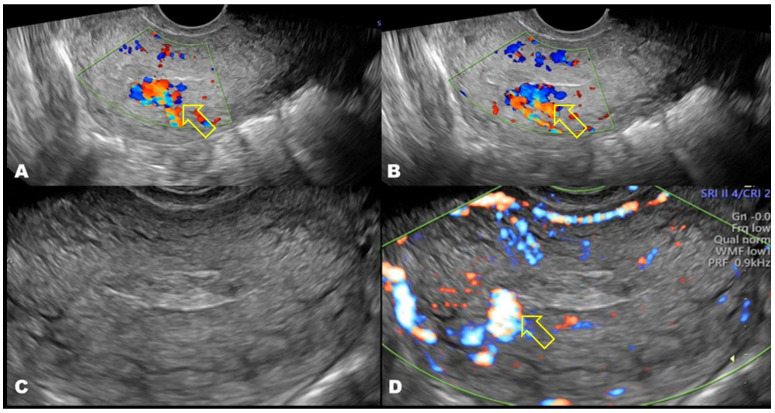
Repeat transvaginal ultrasound with color Doppler demonstrating acquired uterine arteriovenous fistula/malformation. (**A**,**B**) Longitudinal views showing intense myometrial hypervascularization with serpiginous vessels along the posterior uterine wall (indicated by a yellow arrow). (**C**,**D**) Transverse views demonstrating a mosaic pattern of turbulent, high-velocity, low-resistance flow consistent with arteriovenous shunting (indicated by a yellow arrow). AVF, arteriovenous fistula; AVM, arteriovenous malformation.

Nevertheless, AVF-related vascularity may be absent or inconspicuous on initial transvaginal ultrasound examination, particularly in the presence of intrauterine clot or necrotic tissue, potentially delaying diagnosis (Figure 3). In patients with persistent or recurrent uterine bleeding, repeat transvaginal ultrasound with color Doppler may subsequently demonstrate marked myometrial hypervascularization with serpiginous vessels and turbulent, high-velocity, low-resistance flow, fulfilling classic diagnostic criteria for uterine arteriovenous fistula/malformation (Figure 4).

Magnetic resonance imaging (MRI) and computed tomography (CT) angiography may provide complementary anatomic information, particularly in complex or extensive lesions, allowing for assessment of lesion extent and vascular anatomy [4]. Digital subtraction angiography remains the diagnostic gold standard and enables simultaneous therapeutic embolization [5,10,11,12]. Given its invasiveness and radiation exposure, angiography is generally reserved for cases in which AVF/AVM is strongly suspected and interventional treatment is planned.

### 2.5. Management Strategies

Management of acquired uterine AVF/AVM must be individualized, taking into account hemodynamic status, severity and pattern of bleeding, lesion size and vascularity, availability of interventional radiology, and the patient’s reproductive wishes.

#### 2.5.1. Expectant and Medical Management

Small, minimally symptomatic lesions may regress spontaneously, and conservative management with serial Doppler ultrasound follow-up has been reported in selected cases [9]. Medical therapies—including combined oral contraceptives, progestins, gonadotropin-releasing hormone agonists, danazol, and other hormonal regimens—have been used in hemodynamically stable patients when uterine preservation is desired and bleeding is not severe [3,9]. However, evidence supporting medical management remains limited and is largely derived from case reports and small series.

#### 2.5.2. Uterine Artery Embolization

**Figure 5:**
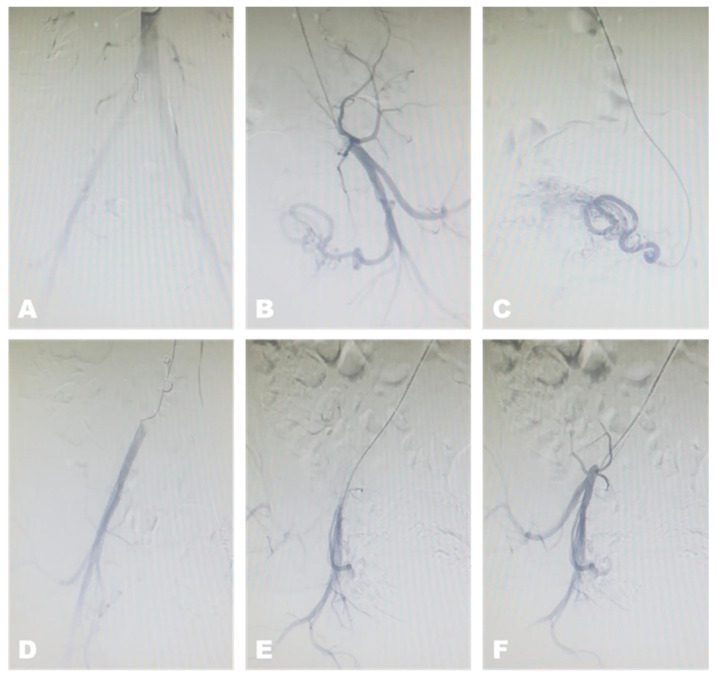
Radiologic aspects of bilateral uterine artery embolization for acquired uterine arteriovenous fistula/malformation. (**A**–**C**) Fluoroscopic visualization of the left uterine artery before embolization, demonstrating abnormal vascularity. (**D**–**F**) Fluoroscopic visualization of the right uterine artery following embolization, showing reduced perfusion and cessation of abnormal flow.

Uterine artery embolization has become the mainstay of fertility-preserving treatment for symptomatic uterine AVF/AVM [5,8,9,10,11,12,17]. Angiographic evaluation allows for confirmation of arteriovenous shunting and enables simultaneous therapeutic embolization of feeding vessels (Figure 5). Various embolic agents, including microparticles, coils, and liquid embolic agents, have been used to occlude feeding vessels and reduce or abolish flow through the malformation [5,10,11,12]. Multiple studies have demonstrated high technical and clinical success rates, with rapid hemorrhage control and low complication rates [5,9,10,11,12]. Repeat embolization may be considered in cases of persistent or recurrent AVF/AVM following initial treatment [5,12].

#### 2.5.3. Surgical Management

Hysterectomy remains a definitive treatment option, particularly for women who have completed childbearing or in cases of failed conservative management or hemodynamic instability [2,5]. Fertility-sparing surgical approaches—including laparoscopic ligation of the uterine artery or ovarian ligament and localized resection of the malformation—have been reported with favorable outcomes in carefully selected patients desiring future fertility [13,14,15].

#### 2.5.4. Hysteroscopic Treatment

**Figure 6:**
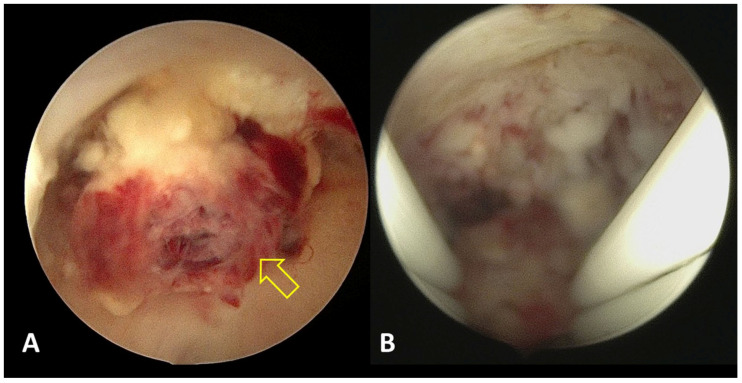
Operative hysteroscopy demonstrating removal of retained intrauterine tissue and coagulation of residual hypervascularity. (**A**) Under direct hysteroscopic visualization, retained intrauterine tissue was identified and completely removed (indicated by a yellow arrow). (**B**) Focal areas of residual hypervascularity adjacent to the cesarean-section scar were selectively coagulated using bipolar electrosurgery.

**Figure 7:**
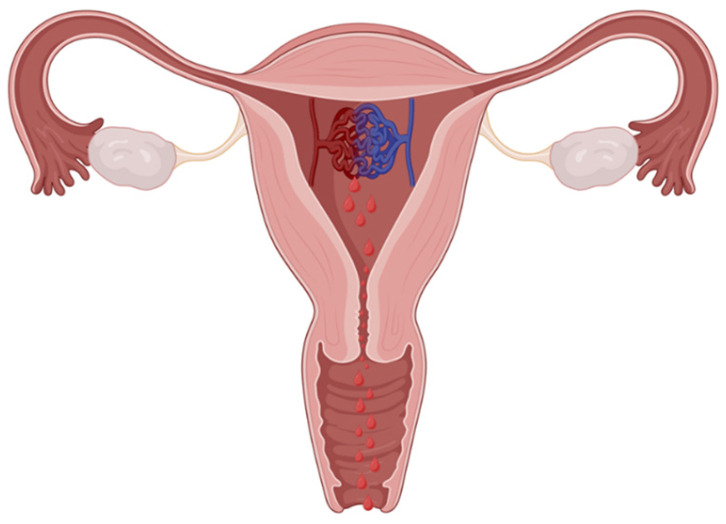
Schematic aspects of AVF/AVM. The abnormal vascular connection within the myometrial structure and the presence of active bleeding are visualized. Created in BioRender. Istrate-Ofițeru, A. (2025) https://BioRender.com/eur0v96. AVF: Arteriovenous fistula; AVM: Arteriovenous malformation.

Hysteroscopic visualization and electrosurgical coagulation or resection of uterine AVF/AVM have been reported as additional minimally invasive options in carefully selected cases. Hysteroscopy allows for direct visualization of the uterine cavity and targeted management of focal lesions projecting into or located immediately beneath the endometrial surface (Figure 6). Several case reports and small series describe successful hysteroscopic management of such lesions, with symptom resolution and preservation of uterine anatomy [15,16]. The anatomical relationship between intrauterine lesions and abnormal myometrial arteriovenous connections is illustrated schematically (Figure 7).

**Figure 8:**
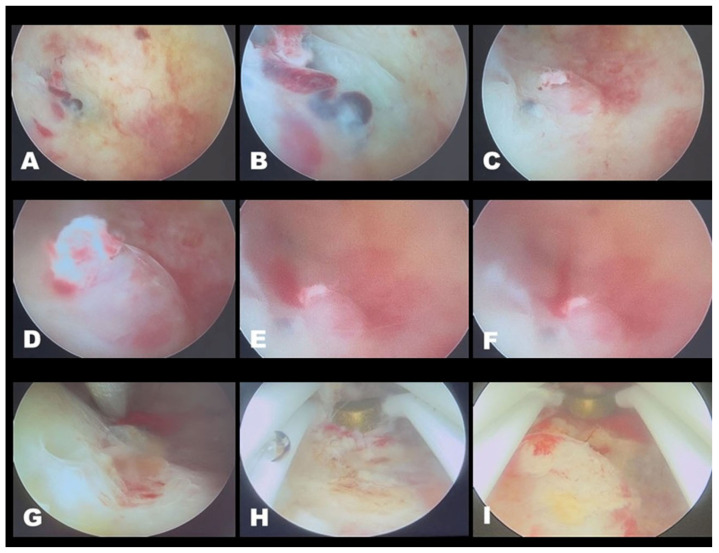
Hysteroscopic management of uterine AVF/AVM. Diagnostic hysteroscopy revealed a pulsatile vascular lesion on the posterior uterine wall, characterized by dilated tortuous vessels with active bleeding, consistent with AVF/AVM. Initial bipolar coagulation was insufficient, requiring targeted electrosurgical coagulation and partial resection using a resectoscopic loop. Targeted electrosurgical coagulation and partial resection resulted in effective control of bleeding. (**A**–**F**) Pulsatile vascular lesion protruding into the uterine cavity. (**G**) Attempted hemostasis using a bipolar coagulation ball. (**H**,**I**) Definitive coagulation using the resectoscopic loop. Abbreviations: AVF—arteriovenous fistula; AVM—arteriovenous malformation.

However, the evidence supporting hysteroscopic management remains limited and is largely derived from case reports and small case series. Consequently, hysteroscopic treatment should be interpreted as a feasible uterus-preserving option in selected, hemodynamically stable patients rather than as a standard or practice-changing approach. In most reported cases, hysteroscopy has been used either as a complementary technique following uterine artery embolization or in highly selected focal lesions with cavity accessibility and immediate availability of hemostatic escalation (Figure 8).

### 2.6. Fertility and Pregnancy Outcomes

Fertility preservation represents a major concern in reproductive-age women diagnosed with uterine AVF/AVM. Although data on reproductive outcomes following conservative management remain limited, available evidence is generally reassuring. Published case series and reviews indicate that many women are able to conceive and achieve term pregnancies after uterine artery embolization or fertility-sparing surgical treatment [8,18]. Nevertheless, some studies have reported an increased risk of placenta-related abnormalities. It should be emphasized that available fertility and obstetric outcome data are limited by small sample sizes, retrospective designs, and heterogeneous follow-up, and therefore should be interpreted with caution.

## 3. Discussion

### 3.1. Diagnostic Considerations in Post-Abortion Uterine AVF/AVM

Acquired uterine arteriovenous fistulas and malformations may present with heterogeneous clinical and imaging features in the post-abortion setting. Persistent or recurrent uterine bleeding following first-trimester pregnancy loss represents a common clinical scenario in which the diagnostic pathway may vary considerably, depending on timing, imaging findings, and clinical suspicion.

Classic Doppler features—such as serpiginous myometrial vessels with turbulent, high-velocity, low-resistance flow—may be evident early after uterine instrumentation, facilitating prompt diagnosis and timely intervention [1,3,4,9]. In some instances, associated findings such as retained trophoblastic tissue may further support the diagnosis of an acquired AVF/AVM and highlight the risk of severe hemorrhage if repeat blind curettage is attempted.

Conversely, initial ultrasound examinations may demonstrate nonspecific intrauterine echogenic material without detectable Doppler flow, delaying recognition of an underlying arteriovenous shunt. In such cases, AVF-related hypervascularity may only become apparent on repeat Doppler evaluation performed in the context of ongoing or recurrent bleeding. Potential explanations include progressive vascular remodeling, hemodynamic changes following initial interventions, or masking of abnormal flow by intrauterine clot. Similar temporal evolution of imaging findings has been described in other reports of pregnancy-related uterine AVF/AVM [3,9].

These observations emphasize a key diagnostic principle: in women with persistent post-abortion bleeding, a single negative Doppler examination does not exclude uterine AVF/AVM. Repeat color and spectral Doppler ultrasound performed by an experienced operator is essential before considering further invasive procedures.

### 3.2. Embolization and Hysteroscopy in Contemporary Management

Current management strategies for uterine AVF/AVM increasingly rely on minimally invasive, fertility-preserving approaches. Uterine artery embolization has emerged as the cornerstone of treatment for symptomatic, high-flow lesions, offering rapid hemorrhage control with high technical and clinical success rates [5,8,9,10,11,12].

Published series and accumulated clinical reports indicate that UAE is particularly effective as first-line therapy in patients presenting with active bleeding or hemodynamic compromise. However, embolization may not be curative in all cases, and persistent or recurrent hypervascularity has been reported following initial treatment [5,12].

In this context, hysteroscopy represents a valuable complementary modality. Direct hysteroscopic visualization allows for identification of focal intrauterine or subendometrial vascular lesions and facilitates targeted electrosurgical coagulation or limited resection. Emerging evidence suggests that hysteroscopic treatment may function not only as an adjunct to UAE but also as a definitive therapeutic option. This approach appears particularly suitable in carefully selected, hemodynamically stable patients with lesions accessible from the uterine cavity [15,16].

From a fertility perspective, both UAE and hysteroscopic management appear to be viable options. UAE has the most robust evidence base, with multiple studies reporting favorable reproductive outcomes [8,9,14,15,18]. Nevertheless, concerns regarding abnormal placentation in subsequent pregnancies persist [19]. Hysteroscopic treatment avoids radiation exposure and may exert less impact on global uterine perfusion, although available data remain limited to small series with encouraging preliminary results [16].

Importantly, while hysteroscopic management has shown promising results in selected reports, the current evidence base is insufficient to support routine changes in clinical practice. Its role should therefore be considered adjunctive and individualized, rather than standardized.

### 3.3. Integrating Clinical Variability into Individualized Management

Clinical experience underscores the heterogeneity of acquired uterine AVF/AVM in terms of presentation, imaging evolution, and response to treatment. Some lesions exhibit early, typical Doppler features and respond predictably to stepwise management strategies, whereas others follow a more complex course characterized by initially equivocal imaging and partial response to embolization.

These observations support an individualized approach to management, guided by clinical stability, lesion accessibility, reproductive goals, and response to initial therapy. While UAE remains highly effective for hemorrhage control, hysteroscopy represents an important component of the therapeutic armamentarium, particularly for focal lesions and in patients for whom minimizing radiation exposure is a priority.

### 3.4. Clinical Gaps and Future Directions

Despite increasing recognition of acquired uterine AVF/AVM, several gaps remain in current knowledge and clinical practice. Standardized diagnostic algorithms integrating clinical assessment, ultrasound, cross-sectional imaging, and angiography are lacking, resulting in variability in diagnostic pathways between institutions.

Similarly, evidence-based criteria guiding selection among expectant management, medical therapy, UAE, hysteroscopic treatment, and hysterectomy remain insufficient. The optimal timing and sequencing of combined approaches, particularly UAE followed by hysteroscopy, have not been clearly defined. In addition, long-term reproductive and obstetric outcomes following conservative management require further investigation through prospective studies with standardized follow-up [8,9,14,15,16,18,19].

Finally, the absence of validated imaging or Doppler predictors of treatment response represents an important area for future research.

In this context, the present review seeks to bridge the gap between existing systematic summaries and day-to-day clinical decision-making in post-abortion care, particularly by emphasizing diagnostic timing, imaging evolution, and individualized use of minimally invasive strategies.

### 3.5. Practical Recommendations for Clinical Practice

Based on available evidence and integrated clinical experience, several practical recommendations can be proposed:

1. Maintain a high index of suspicion for uterine AVF/AVM in women with persistent or recurrent bleeding following aspiration abortion, curettage, or cesarean delivery.
2. Use color and spectral Doppler ultrasound systematically when evaluating post-pregnancy bleeding, as grayscale imaging alone is insufficient for diagnosis.
3. Repeat Doppler ultrasound in symptomatic patients with an initial negative or equivocal examination, particularly before performing repeat blind curettage.
4. Avoid repeat curettage when AVF/AVM is suspected, as it may precipitate severe hemorrhage [3,8,20,21,22].
5. Consider uterine artery embolization as first-line treatment for hemodynamically significant lesions or active heavy bleeding when interventional radiology is available [5,9,10,11,12].
6. Consider hysteroscopic treatment in carefully selected, hemodynamically stable patients with focal lesions accessible from the uterine cavity, particularly as an adjunct after uterine artery embolization or in settings where immediate escalation of care is available, acknowledging the limited level of supporting evidence [15,16,17,23].
7. Whenever possible, manage these patients in centers with combined expertise in interventional radiology and minimally invasive gynecologic surgery, enabling individualized, multimodal care.

## 4. Conclusions

Acquired uterine arteriovenous fistulas and malformations are rare but increasingly recognized causes of abnormal uterine bleeding in reproductive-age women, particularly in postpartum and post-abortion settings. They should be considered in any patient presenting with persistent or recurrent bleeding following uterine instrumentation or pregnancy loss.

Color and spectral Doppler ultrasonography are central to diagnosis; however, imaging findings may evolve over time, and a single negative Doppler examination does not exclude uterine AVF/AVM when clinical suspicion remains high. MRI, computed tomography angiography, and digital subtraction angiography provide additional anatomic detail and are valuable in selected complex cases, particularly when interventional treatment is being considered.

Clinical experience and available evidence converge on a key principle: individualized, minimally invasive management can effectively control hemorrhage while preserving the uterus. Uterine artery embolization remains the cornerstone of treatment for many symptomatic lesions, whereas hysteroscopic resection or coagulation has emerged as a valuable complementary option in carefully selected cases.

Future research should focus on standardizing diagnostic algorithms, refining selection criteria for each treatment modality, and generating robust long-term data on fertility and pregnancy outcomes following conservative management. Until such evidence becomes available, multidisciplinary care in experienced centers and careful, individualized decision-making remain essential to optimizing outcomes in this uncommon but potentially life-threatening condition.

## Data Availability

All data presented here are available from the authors upon reasonable request.

## Abbreviations

The following abbreviations are used in this manuscript:

AVF	arteriovenous fistula
AVM	arteriovenous malformation
CT	computed tomography
DSA	digital subtraction angiography
EMV	enhanced myometrial vascularity
GnRH	gonadotropin-releasing hormone
MRI	magnetic resonance imaging
PAIRS	Procedural Abortion Incident Reporting and Surveillance
RPOC	retained products of conception
TVUS	transvaginal ultrasound
UAE	uterine artery embolization
